# Chlorate of Potassa—Some of Its Uses

**Published:** 1861-12

**Authors:** S. R. Percy

**Affiliations:** Professor of Mat. Med. and Ther., in the N. Y. Med. Coll. and Charity Hospital


### Selections.

### CHLORATE OF POTASSA—SOME OF ITS USES.

An Extract from a Lecture, by S. R. Percy, M. D., Professor of Mat. Med. and Ther., in the N. Y. Med. Coll. and Charity Hospital. Reprinted from the *Am. Med. Times.*

The diseases in which of late years this remedy has been most successfully employed, are diphtheria, and ulcerative or gangrenous sore throat. In diphtheria, it has been probably used more largely by Professor Jacobi than by anybody else, though latterly he has used $NaO, ClO_5$, in preference to $KO, ClO_5$, not only on account of its greater solubility, but because he informs me, it is more mild in its effects. In these diseases, it is used not only as a wash and gargle to the mouth and throat, but it is also taken internally in considerable quantities. Whether it acts, as has been so repeatedly asserted, by giving up a portion of its oxygen to the blood, I will, after what you have heard, leave you to decide; but we do know that it is not all decomposed, as some of it is found in the urine, saliva, fæces, and sweat unchanged. Professor Tully and other eminent men, reject the theory that it imparts free oxygen to the blood. It is my opinion that it acts rather by its unity as a saline, possessing its own peculiar action, than by decomposition. That it produces a florid color of the blood without being decomposed, we have abundant proof. There are symptoms in typhoid fever that are much relieved by its careful exhibition. When, in this disease, the urine is found to be scanty and high colored, with the brain dull, owing to this scanty secretion, this remedy given in moderate doses, freely diluted with water, for twenty-four or forty-eight hours, will generally be found to give great relief by its restorative and diuretic action. I have seen it of great service in cases of scurvy, where the gums and lips and skin were of a livid color; and it may have acted in the manner that Dr. Garrod asserts that these salts act—by supplying potash to the blood; and if it acted in this way, it must have been decomposed. It did not as a free and efficient diuretic. In mercurial stomatitis it is a very successful remedy, both as a wash, and when taken internally, but it is not by any means a specific. In syphilis, it has been found of but little service. On the whole, it is perhaps the best remedy we have for buccal inflammations and for ozœna.

It is an interesting question to ask, but one which has yet to be answered, What is the difference in the therapeutic effects of KO, $ClO_5$ (chlorate of potash), NaO, $NO_5$, (nitrate of soda), NaO, $ClO_5$, (chlorate of soda), and KO, $NO_5$, (nitrate of potash)?

When taken in any considerable dose, it depresses the heart's action, frequently reducing the pulse twenty or more beats in the minute; this it does by its refrigerant and antiphlogistic powers. That it possesses tonic properties, as claimed by Fountain, and others before him, is entirely out of the question, excepting so far as its base may serve as a restorative hæmatic.

In what manner does KO, $ClO_5$ act, when taken in small or medicinal doses; and what are its effects and modus operandi when taken in inordinate doses? We have seen by the experiments of O'Shaughnessy, that it brightens the color of the blood; that it revived animals, when poisoned by medicines that acted on that fluid; and we have seen that a part of it, at least, traversed the system and passed from it in the same state in which it entered it, thus producing its effects by its unity, not by decomposition. We have seen, again, by the experiments that I related to you, that in certain states of the system, decomposition does not take place, and that both base and acid are used for the reparation of tissues, and that this decomposition takes place only so long as it is needed as a restorative hæmatic; and that as soon as the system is saturated with it, it appears in the urine, and after awhile passes off, also, by the bowels. We learn, also, from the large experience of Professor Jacobi, in the Dispensary, that it is the best remedy we at present possess for the various forms of inflammatory sore mouth, both when used as a wash, and given internally; and that, though it frequently fails to afford relief, it is one of the best remedies in diphtheritic exudations, and in mercurial ptyalism. The latter fact is verified by the previous experiments of Herpin, Blache, and Ricord. That it is no *specific* in phthisis, and, in fact, that it frequently does injury in that disease, we learn from the observations of Köhler, Flint, Gay, and others.

As to its effects in large doses, we see that it may even be fatal, and that death may be produced by its action on the kidneys, and upon the stomach and bowels. In the cases related by Tully, in which one ounce was taken at a dose, there was a great uniformity in the symptoms produced. In one instance, in one hour after taking an ounce, the pulse fell from seventy-two to fifty-six, and was considerably smaller and weaker, and in five hours the pulse had fallen to thirty-six. It produced, also, a severe, heavy, and oppressive pain in the stomach and bowels, with free and painful alvine evacuations. Even on the second and third days this severe and oppressive pain was experienced, and was only relieved by large doses of clear brandy, and excessively large doses of opium. If the opium was omitted, the pain returned, of a lancinating and sore character, with intolerance of the slightest pressure. The violence of the symptoms passed off after a copious diuresis, but it left the person with flatulence and other dyspeptic symptoms, which lasted for some time. The treatment in these large doses should be large quantities of warm flaxseed tea, the hot bath, and blistering with ammonia over the kidneys, with large doses of opium and ipecac.

Professor Jacobi has kindly furnished me a most elaborate culling of the German journals, on the effects and uses of this salt, and I am very sorry that I have not time to present these interesting notes to you, especially as Professor Jacobi's own very extensive experience with the salt, has enabled him to make an excellent selection of what was worth mentioning. I hope to give you a collation from these notes at a future time. Dr. Jacobi's individual experience with this remedy is perhaps more extended than that of any other person amongst us; for in the German Dispensary, his clinic and private practice, I find he has notes of its administration in about two thousand cases, and, as I have before said, he uses it to a great extent, and with good success, both internally, and as a gargle and wash, in the various forms of stomatitis. In mercurial stomatitis, Dr. Jacobi amply verifies Ricord's experience, and has met with almost unvarying success in keeping off salivation. From the first day of a mercurial treatment for syphilis, he has given $KO, ClO_5$ or $NaO, ClO_5$ in drachm doses, without any abatement of the effects of the Hg (mercury), and no salivation, even where the Hg has been given, from four to six weeks. Dr. Jacobi informs me that about three years ago, he took two ounces of $KO, ClO_5$ in divided doses during two days. During the first day, there was nausea during the whole time, the urine was increased, free salivation all day, slight diarrhœa, and loss of appetite. During the second day, the nausea was more intense, a constant spitting, a very sore feeling in the intestines diarrhœa increased, with general uneasiness and irritability; he was not fully over its effects for several days. But Dr. Jacobi says, there are but very few to whom he would give this dose. As to its detection in the secretions, Isambus found it in the saliva five minutes after taking it, in the urine in ten minutes; he found it, also, in the milk, tears, nasal mucus, and perspiration, but not in the fæces or semen.

